# Investigating the biochemical association of gestational diabetes mellitus with dyslipidemia and hemoglobin

**DOI:** 10.3389/fmed.2023.1242939

**Published:** 2023-10-26

**Authors:** Muhammad Sajid Hamid Akash, Sibgha Noureen, Kanwal Rehman, Ahmed Nadeem, Mohsin Abbas Khan

**Affiliations:** ^1^Department of Pharmaceutical Chemistry, Government College University, Faisalabad, Pakistan; ^2^Department of Pharmacy, University of Chenab, Gujrat, Pakistan; ^3^Department of Pharmacy, The Women University, Multan, Pakistan; ^4^Department of Pharmacology and Toxicology, College of Pharmacy, King Saud University, Riyadh, Saudi Arabia; ^5^School of Cancer and Pharmaceutical Science, Faculty of Life Science and Medicine, King's College London, London, United Kingdom; ^6^Department of Pharmaceutical Chemistry, The Islamia University of Bahawalpur, Bahawalpur, Pakistan

**Keywords:** gestational diabetes mellitus, biochemical association, hemoglobin level, lipid profile, glycated hemoglobin

## Abstract

**Aims:**

To investigate the biochemical correlation of hemoglobin (Hb), dyslipidemia, and HbA1c with gestational diabetes mellitus (GDM).

**Background:**

GDM is a condition that develops during pregnancy and is characterized by high blood sugar levels. Biochemical parameters such as hemoglobin (Hb), dyslipidemia, and HbA1c have been implicated in the development of GDM. Understanding the correlation between these biochemical parameters and GDM can provide insights into the underlying mechanisms and potential diagnostic markers for the condition.

**Objective:**

The objective of this study was to evaluate the correlation of various biochemical parameters, including Hb, dyslipidemia, and HbA1c, in pregnant women with and without GDM.

**Method:**

A cross-sectional study design was used. Pregnant females attending a tertiary care hospital in Faisalabad between September 1st, 2021, and June 25th, 2022, were included in the study. The participants were divided into two groups: those with GDM (GDM group) and those without GDM (non-GDM group). Blood glucose, Hb, and lipid levels were compared between the two groups using statistical tests, including chi-square, independent sample *t*-test, and Pearson’s correlation.

**Result:**

Out of the 500 participants, 261 were in the 2nd trimester and 239 in the 3rd trimester. Maternal age showed a significant difference between the GDM and non-GDM groups. The levels of Hb, TC, HDL, LDL, and HbA1c significantly differed (*p* < 0.05) between the two groups. TC (*r* = 0.397), TG (*r* = 0.290), and LDL (*r* = 0.509) showed a statistically significant and moderately positive correlation with GDM. HDL (*r* = −0.394) and Hb (*r* = −0.294) showed a moderate negative correlation with GDM.

**Conclusion:**

Increased levels of HbA1c, TC, and LDL, along with decreased levels of HDL and Hb, were identified as contributing factors to GDM. The levels of TC, TG, and LDL were positively correlated with GDM, while HDL and Hb were negatively correlated. The findings of this study suggest that monitoring and managing hemoglobin, dyslipidemia, and HbA1c levels during pregnancy may be important in identifying and potentially preventing or managing GDM. Further research is needed to explore the underlying mechanisms and potential interventions targeting these biochemical parameters in relation to GDM.

## 1. Introduction

Gestational diabetes mellitus (GDM) is a condition of glucose intolerance during pregnancy (1). It usually develops in the second or third trimester and may resolve after delivery. GDM results from insufficient insulin production to regulate blood glucose levels during pregnancy. The prevalence of GDM worldwide ranges from <1 to 28%, with an 8% prevalence in Pakistan (2). According to recent estimates, approximately 18.6 million women have experienced various hyperglycemic conditions, with 18.6% of them suffering from GDM (3). The prevalence of GDM is higher in the South Asian region, with a recorded prevalence of 24.2% (4). The WHO and International Association of the Diabetes and Pregnancy Study Groups criteria (IADPSGC) note that neonatal and maternal complications such as macrosomia, respiratory distress, perinatal mortality, shoulder dystocia, childhood obesity, and neonatal hypoglycemia may arise in women with GDM (5, 6).

Abnormal glycolysis may have a negative impact on lipid metabolism. Placental hormones such as estrogen, prolactin, and human placental lactogen are significant contributors to insulin resistance (IR) and maternal adiposity, resulting in atherogenic dyslipidemia (AD) (7). Hyper-dyslipidemia during the latter half of pregnancy is considered a necessary mechanism for providing nutrients and metabolic fuel to the fetus (8). A primary risk factor for developing gestational diabetes (GDM) and change in lipid metabolism is Maternal obesity (9, 10). A prior study demonstrated that overweight women with increased triglycerides are at a greater risk of developing GDM than lean women with higher HDL (9). Additional risk factors for dyslipidemia include activity level, smoking status, age, blood glucose level, gender, and overall patient health (11). Pregnancy and GDM have a cumulative effect on the lipid profile. In diabetes, HbA1c (glycated hemoglobin) is a commonly used indicator for measuring blood sugar levels, reporting the average plasma glucose over approximately 8–10 weeks. Elevated HbA1c disrupts the lipid profile, with a 1% increase in HbA1c resulting in an 18% increase in the lipid profile and a higher risk of several cardiovascular diseases (12). During pregnancy, dyslipidemia characterized by elevated triglycerides (TG) or total cholesterol (TC) may increase the risk of preterm delivery, the second most common cause of death in children under 5 years of age (13). Moreover, it raises the risk of metabolic derangement later in life.

Hemoglobin is a protein that carries oxygen and circulates in the bloodstream, supplying oxygen to bodily tissues. During pregnancy, there is an increased demand for red blood cells, which in turn increases the demand for iron and vitamins. This increased demand for iron and vitamins affects the level of hemoglobin. Iron deficiency may result in reduced hemoglobin production, leading to iron deficiency anemia, which is a common cause of anemia during pregnancy. According to the World Health Organization (WHO), a woman is considered anemic if her hemoglobin level is less than 11 g/dL (14). Consuming iron-rich foods or taking iron supplements can help compensate for the iron deficiency and meet the body’s needs. However, increasing iron levels can have adverse effects: a previous study has shown that the prevalence of GDM is strongly linked with high levels of iron in the blood (15).

This study aims to investigate the significant correlation between Hb levels, dyslipidemia, and HbA1c with GDM. To the best of our knowledge, this is the first report on the correlation of GDM with various biochemical parameters (including Hb, HbA1c, and lipid profile) in a single population in Pakistan.

## 2. Materials and methods

### 2.1. Study design and location

**Figure 1:**
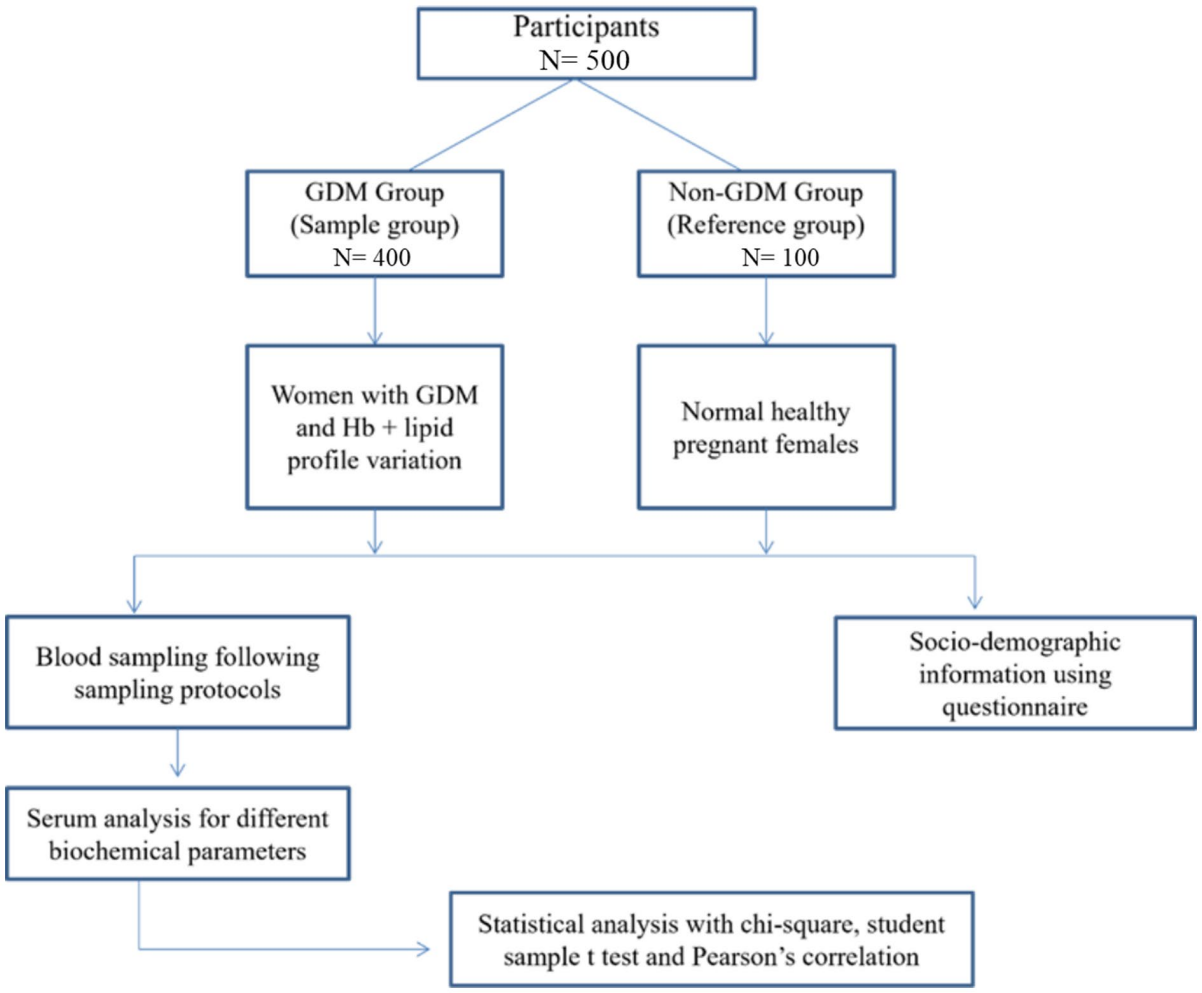
Study flow diagram.

This prospective cross-sectional study recruited 500 pregnant women attending Allied Hospital Faisalabad, Pakistan, between September 1st, 2021, and June 25th, 2022. Participants were divided into two groups: a reference group consisting of 100 normal, healthy pregnant women, and a study group consisting of 400 pregnant women with GDM and variable Hb and lipid profiles. Informed consent was taken from all participants after explaining the study’s objectives to them. Figure 1 provides a study flow diagram.

### 2.2. Data collection

We used a structured data collection form to extract relevant information from study participants. Patient names, ages, BMIs, histories of chronic diseases, trimesters, and gravidity were included in the sociodemographic information. A questionnaire, both in soft and hard form and in the English language, was available to extract demographic information through patient interviews. However, laboratory data were extracted from patients’ files.

### 2.3. Selection criteria

#### 2.3.1. Inclusion criteria

This study included pregnant women with unstable lipid profiles and GDM, as well as healthy pregnant women in their second and third trimesters, and pregnant women with varying Hb concentrations and GDM.

#### 2.3.2. Exclusion criteria

This study excluded non-pregnant females, pregnant females with a family history of diabetes, pregnant females with chronic illnesses, and pregnant females in their first trimester.

### 2.4. Biochemical analysis

Blood samples were taken from the patients to measure the concentration of blood glucose, Hb, and lipid parameters. Before taking blood samples, blood pressure was checked using a digital BP apparatus (Certeza 405). Additionally, oxygen saturation was measured using an oximeter. Various enzymatic biochemical assays and strip methods (for blood glucose) were used to analyze the blood samples. In common practice, screening for GDM is typically conducted between the 24th and 28th week of gestation because GDM often manifests at this stage. Glucose screening is carried out through either the Glucose Challenge Test or the Oral Glucose Tolerance Test. The diagnostic criteria for GDM can vary slightly from one hospital to another depending on the region and the guidelines provided to healthcare providers. Typically, the diagnostic criteria are based on the following thresholds:

- Fasting blood glucose level > 95 mg/dL or ≤ 5.27 mmol/L (Before a meal).
- Blood glucose level > 140 mg/dL or ≤ 7.8 mmol/L (1 h after a meal).
- Blood glucose level > 120 mg/dL or ≤ 6.7 mmol/L (2 h after a meal).

### 2.5. Statistical analysis

The data were analyzed using IBM-SPSS Statistics version 21. Continuous data were expressed as mean with standard deviation and compared using an independent sample *t*-test. Categorical data were expressed as frequency with percentages and compared using a chi-square test. The correlations among variables were ascertained using Pearson’s correlation test. A *p*-value of < 0.05 was considered statistically significant throughout the analysis.

## 3. Results

### 3.1. Demographics and clinical characteristics of participants

A total of 500 participants were recruited for this study, consisting of 400 pregnant females with gestational diabetes mellitus (GDM) and 100 normal, healthy pregnant females without GDM. Of the total participants, 261 females (52.1%) were in the second trimester, while 239 (47.8%) were in the third trimester. About 338 females (67.6%) were primigravida, while 162 females (32.3%) were multigravida or secundigravida. Statistical analysis using the chi-square test revealed significant results (*p* < 0.05) for two parameters, age and BP. The detailed social-demographics and the clinical characteristics of the study participants are presented in Table 1.

**Table 1:** Demographics and clinical characteristics of study participants.

Sr. #	Parameters	Categories	Frequency (%) (*N* = 500)	GDM *n* = 400	Non-GDM *n* = 100	*p*-value*
1	Age	20–30 years	127 (25.3%)	61 (15.2%)	43 (43%)	0.04
		31–40 years	204 (40.8%)	192 (47%)	31 (31%)	
		41–50 years	169 (33.8%)	147 (36.7%)	26 (26%)	
3	BMI	Normal body weight (18.5–24.9 kg/m^2^)	233 (46.6%)	175 (43.7%)	52 (52%)	0.097
		Obesity (≥30 kg/m^2^)	21 (4.2%)	21 (5.2%)	4 (4%)	
		Overweight (25–29.9 kg/m^2^)	225 (45%)	204 (51%)	32 (32%)	
		Underweight (<18.5 kg/m^2^)	21 (4.2%)	0	12 (12%)	
4	Trimester	2nd trimester	261 (52.1%)	176 (44%)	66 (66%)	0.089
		3rd trimester	239 (47.8%)	224 (56%)	34 (34%)	
5	Gravidity	Primigravida	338 (67.60%)	277 (69.3%)	66 (66%)	0.761
		Multigravida	162 (32.3%)	123 (30.8%)	34 (34%)	
6	Blood pressure	120/80 mm Hg (normal BP)	275 (54.9%)	220 (55%)	56 (56%)	0.003
		140/90 mm Hg (high BP)	169 (33.8%)	169 (42.3%)	20 (20%)	
		90/60 mm Hg (low BP)	56 (11.2%)	11 (2.7%)	24 (24%)	
7	Oxygen saturation	92.9–99.3%	261 (52.1%)	175 (43.8%)	66 (66%)	0.89
		93.4–98.5%	239 (47.8%)	225 (56.3%)	34 (34%)	

The *p* values were estimated using the Cross-tabulation method by using Chi-square or Fischer Exact test.

### 3.2. Comparison of biochemical parameters between patients with and without GDM

The inter-group comparison revealed that increased age, a low prevalence of Hb and HDL levels, and high levels of TC, LDL, and HbA1c were significantly associated with GDM. However, BMI and TG levels were equally distributed across the two groups (refer to Table 2).

**Table 2:** Comparison of biochemical parameters among females with and without GDM.

Parameters	GDM (*n* = 400) Mean ± SD	Non-GDM (*n* = 100) Mean ± SD	*p*-value*
Age (years)	33.06 ± 7.3	29.0 ± 8.7	**0.042**
BMI (kg/m^2^)	2.6 ± 0.57	2.30 ± 0.73	0.068
Hb level (g/dL)	0.60 ± 0.86	1.42 ± 0.85	**<0.001**
TC (mg/dL)	182.0 ± 36.14	143.0 ± 35.2	**<0.001**
TGs (mg/dL)	173.06 ± 55.0	155.15 ± 16.52	0.112
HDL (mg/dL)	46.66 ± 9.10	54.88 ± 8.5	**<0.001**
LDL (mg/dL)	126.44 ± 25.99	105.1 ± 14.98	**<0.001**
HbA1c (%)	6.20 ± 0.62	5.30 ± 0.47	**<0.001**

*The *p* values were estimated using the Student-*t* test.

SD, Standard Deviation; BMI, Body mass index; Hb, Hemoglobin; TC, Total Cholesterol; TGs, Triglycerides; HDL, High-Density Lipoproteins; LDL, Low-Density Lipoproteins; HbA1c, Glycated hemoglobin.

*p* value stands for statistically significant value with *p* < 0.05. All the bold values showed significant results.

### 3.3. Correlation of biochemical parameters with glycated hemoglobin (HbA1c)

Our analysis revealed a positive correlation between total cholesterol (*r* = 0.397), triglycerides (*r* = 0.290), and LDL (*r* = 0.509), and a negative correlation between HDL (*r* = −0.394) and Hb (*r* = −0.294) with glycated hemoglobin (Table 3).

**Table 3:** Correlation of different biochemical parameters with glycated hemoglobin.

Parameters	*r* value	*p*-value
Age	0.15	0.192
Body mass index (BMI)	0.75	0.537
Hemoglobin (Hb)	−0.294	**0.013**
Total cholesterol (TC)	0.397	**0.001**
Triglycerides (TGs)	0.290	**0.014**
Low density lipoprotein (LDL)	0.509	**<0.001**
High density lipoprotein (HDL)	−0.394	**0.001**

*r*, Pearson correlation.

*p* value stands for statistically significant value with *p* < 0.05. All the bold values showed significant results.

## 4. Discussion

This study aims to investigate the correlation between hemoglobin (Hb) levels and lipid profiles with gestational diabetes mellitus (GDM) in a case-control manner. Previous studies have typically examined the correlation between Hb or lipid profile and GDM in discrete cohorts, rather than in a single population. Our findings demonstrate that age is a contributing factor for GDM, as it differed significantly between the two groups (*p* < 0.05). These results align with other studies that have found advanced maternal age, in addition to other maternal characteristics, to be a predisposing factor for GDM (16, 17). A meta-analysis of approximately 120 million participants also reported maternal age as a risk factor for GDM (11). The meta-analysis included 24 studies, which reported a linear relationship between maternal age and GDM (P trend <0.001). Furthermore, it revealed that for Asian women, every one-year increase in maternal age results in a 12.74% increase in the risk of developing GDM.

There was a statistically significant difference in Hb levels between the two study populations (*p* < 0.05). Women with GDM had lower levels of iron in their blood compared to the non-GDM group. During gestation, many women experience lower Hb levels in their blood and may take iron supplements to compensate for iron deficiency. Variations in iron levels affect maternal glucose homeostasis and may lead to the development of GDM (18). Our findings showed a mild negative correlation (*r* = −0.294) between Hb and HbA1c. This contravenes a study from Korea, which reported an association between higher Hb levels and GDM (19). Most previous studies have not examined the correlation of Hb and lipid profile with GDM in a single population, which makes our findings particularly noteworthy. Additionally, our results indicate that advanced maternal age is a significant contributing factor for GDM, which is consistent with other studies (11, 16, 17).

HbA1c is an indicator of the average blood glucose levels for 3–4 months and is considered a reliable measure of the sugar level of diabetic patients. In our study, we found statistically significant differences in HbA1c levels between the two groups (*p* < 0.05). An independent sample *t*-test was used to compare the mean and standard deviation of HbA1c between the GDM and non-GDM groups. Our findings are consistent with a previous study that reported higher HbA1c levels in the GDM group compared to the non-GDM group and a positive linear correlation between HbA1c and blood glucose levels (*p* < 0.001) (20).

Our analysis revealed significant differences in TC, HDL, and LDL between the GDM and non-GDM groups (*p* < 0.05). These findings are consistent with the results of a previous hospital-based study that showed significant differences in lipid parameters between the two groups (*p* < 0.05) (21). However, our results contradict those of a previous study from Nigeria, which found no significant association between lipid profiles and GDM status (22).

Pearson’s correlation was used to estimate the correlation between lipid profile and HbA1C. Statistically significant results were found for all four lipid parameters: TC, HDL, LDL, and TG, with glycated hemoglobin. All lipid parameters, except HDL (which showed a negative correlation), were positively correlated with HbA1C. The negative correlation between HDL and HbA1C indicates that an increase in HbA1C levels may lead to a decrease in good cholesterol, resulting in various complications. In gestational diabetes mellitus, an increase in maternal lipid profile may cause the accumulation of fetal fat as it increases the activity of lipoprotein lipase, which results in a higher rate of fatty acid transfer through the placenta (8). Our findings are consistent with previous studies conducted in China, which identified the lipid profile as an independent risk factor for GDM (23, 24). While changes in lipid profile during pregnancy are considered normal, the risk of blood lipid disorders is higher in gestational diabetes mellitus, and monitoring of the lipid profile during early pregnancy is recommended (25).

GDM represents a prevalent pregnancy complication, often manifesting between the 24th and 28th gestational week (26). This condition engenders diverse maternal and fetal complications. Maternally, it may contribute to recurrent vaginal infections, heightening the risk of cesarean section, and inducing polyhydramnios (27). Conversely, the fetus can experience macrosomia and various metabolic perturbations. Our study offers indispensable insights into the multifactorial determinants of GDM and underscores the significance of vigilant maternal health surveillance during pregnancy. These revelations carry profound implications for the clinical management and risk stratification of expectant mothers grappling with GDM. Primarily, the cornerstone of GDM management entails dietary modifications and structured exercise regimens (28). Remarkably, our research corroborates that a substantial proportion of pregnant women with GDM, ranging between 70 and 90%, can achieve glycemic control through non-pharmacological interventions exclusively (29). Furthermore, the National Institute for Health and Care Excellence (NICE) formally advocates for exercise, dietary adjustments, and lifestyle modifications as crucial non-pharmacological interventions for GDM (30). Supplementary to these measures, the incorporation of Myo-inositol within a tailored therapeutic regimen, which may encompass insulin therapy, oral hypoglycemic agents, and metformin, can confer additional benefits to the patient (31). Furthermore, owing to the inherent maternal and fetal risks associated with GDM, healthcare professionals have a professional obligation to engage in comprehensive discussions with patients. These dialogs should encompass an elucidation of the procedural risks and benefits, as well as potential alternatives (32). The medical community is increasingly embracing the concept of personalized medicine (33), necessitating a keen focus on individualized care and patient priorities (34). In a broader context, emerging studies have illuminated the potential of artificial intelligence, centered around human expertise, to augment and enhance the capabilities of healthcare professionals concerning reproductive medical issues (35). The correlations unearthed by our study advocate for the vigilant monitoring and management of hemoglobin, dyslipidemia, and HbA1c levels during pregnancy. This proactive approach may serve as a pivotal tool in the early identification and prospective prevention or management of GDM.

The limitations of this study include the impact of selection bias, confounding factors, and a heterogeneous population. Future studies should address these limitations in their study design. Nonetheless, this study provides valuable guidance for healthcare professionals in identifying factors associated with GDM, which can lead to personalized and prioritized care for patients.

## 5. Recommendations for healthcare professionals

Certainly, based on our study’s findings, we can provide the following possible recommendations for healthcare professionals who are involved in the care and management of pregnant women with GDM: Conduct a thorough risk assessment for each pregnant woman, considering factors such as hemoglobin levels, dyslipidemia, and HbA1c during pregnancy. Utilize these risk assessments to stratify patients into low, moderate, or high-risk categories. Tailor care plans based on the individual risk profiles of pregnant women with GDM. Customize dietary recommendations and exercise programs to match each patient’s unique needs and risk factors. Emphasize the importance of non-pharmacological interventions, including diet and exercise, as first-line treatments for GDM. Educate the patients about the benefits of lifestyle modifications and their potential to achieve glycemic control without medication. Engage in open and patient-centered discussions with pregnant women diagnosed with GDM. Explain the risks and benefits of different treatment options, allowing the patient to make informed decisions about their care. Provide alternatives and address any concerns or questions the patient may have regarding their treatment plan. Implement regular monitoring of glycemic control, hemoglobin levels, and lipid profiles throughout the pregnancy to track progress and make necessary adjustments to the care plan. Adjust the care plan as needed to ensure that glycemic targets are met and that the patient remains within their individualized risk category. Stay current with the latest research and clinical guidelines related to GDM management. Be prepared to adapt care plans and recommendations based on evolving evidence and best practices. Collaborate with other healthcare professionals, such as dietitians, obstetricians, and endocrinologists, to provide comprehensive care for pregnant women with GDM. Ensure that the patient receives holistic care addressing both diabetes management and overall maternal and fetal health.

These recommendations are intended to guide the healthcare professionals in optimizing the care and management of pregnant women with GDM. By incorporating these findings into clinical practice, healthcare providers can contribute to improved outcomes and the well-being of both mothers and their babies.

## 6. Conclusion

This study provides essential insights into the factors associated with GDM and the importance of monitoring maternal health during pregnancy. These findings may have crucial implications for clinical management and risk stratification for pregnant women with GDM. However, the study is limited by selection bias, confounders, and heterogeneous population, which should be considered while interpreting the results. Future studies should take into account these limitations and continue to investigate factors associated with GDM to improve the care of pregnant women and their infants.

## Data availability statement

The raw data supporting the conclusions of this article will be made available by the authors, without undue reservation.

## Ethics statement

This research was approved by the Ethical Review Committee of GCUF (Ref. No. GCUF/ERC/37). This research involved human participants and all the protocols about human participants were followed. The studies were conducted in accordance with the local legislation and institutional requirements. The participants provided their written informed consent to participate in this study.

## Author contributions

MA contributed to project administration, conceptualization, study design, and manuscript writing. SN contributed to formal analysis, sample detection, data processing, and literature search. KR contributed to conceptualization, study design, data curation, and revising it critically for intellectual content. AN contributed to funding acquisition and recourses management. MK contributed to manuscript review. All authors contributed to the article and approved the submitted version.
